# Dynamics of a Panzootic: Genomic Insights, Host Range, and Epidemiology of the Highly Pathogenic Avian Influenza A(H5N1) Clade 2.3.4.4b in the United States

**DOI:** 10.3390/v17030312

**Published:** 2025-02-25

**Authors:** Mohammad Jawad Jahid, Jacqueline M. Nolting

**Affiliations:** College of Veterinary Medicine, The Ohio State University, Columbus, OH 43210, USA

**Keywords:** HPAI A(H5), clade 2.3.4.4b, gs/GD, wild birds, wild mammals, public health, food security

## Abstract

In late 2021, Eurasian-lineage highly pathogenic avian influenza (HPAI) A(H5N1) viruses from HA clade 2.3.4.4b were first detected in the United States. These viruses have caused severe morbidity and mortality in poultry and have been detected in numerous wild and domestic animals, including cows and humans. Notably, infected cows transmitted the virus to cats, causing extreme pathogenicity and death. While human-to-human spread of the virus has not been recorded, efficient transmission of the bovine-origin virus has also led to extreme pathogenicity and death in ferret models. Recently, markers in PB2 (E627K) and HA (E186D, Q222H), indicating mammalian adaptation mutations, were detected in an H5N1-infected patient manifesting critical illness in Canada. These, combined with instances of interspecies spread of the virus, have raised global public health concerns. This could highlight the potential for the virus to successfully adapt to mammals, posing a serious risk of a global outbreak. A One Health approach is, thereby, necessary to monitor and control the outbreak. This review aims to analyze the epidemiology, transmission, and ecological impacts of HPAI A(H5N1) clade 2.3.4.4b in the U.S., identify knowledge gaps, and inform strategies for effective outbreak management and mitigation.

## 1. HPAI A(H5N1) Has Been a Global Threat

Among the current global threats from infectious diseases to animals and public health, influenza viruses of the highly pathogenic avian influenza (HPAI) subtypes H5 and H7 perhaps represent the highest risk [1,2,3,4]. Since their initial detection in a goose in Guangdong, China, in 1996, the A(H5Nx) subtype of HPAI has caused multiple intercontinental outbreaks. Between 2005 and 2023, outbreaks of HPAI viruses have led to the death or extermination of over 547 million poultry worldwide, with an unparalleled peak of 146 million in 2022 across 84 countries and territories [5]. Although avian influenza A virus outbreaks primarily occur within animal populations, their ongoing risks to humans remain significant [6]. The H1N1 pandemics of 1918 and 2009, infecting millions of people around the world, serve as clear examples of the ability of these viruses to cause devastating infections in humans [3]. The threat is further emphasized by the World Health Organization (WHO) report documenting a total of 878 human cases and 458 deaths (52% case fatality rate) associated with the HPAI A(H5N1) strain, spanning across 23 countries between January 2003 and July 2023 [7].

Since 2020, a variant of the goose/Guangdong-lineage of HPAI A(H5N1), belonging to clade 2.3.4.4b, has been detected in wild birds and has caused unusual mortalities in poultry and wild mammals across different countries [8]. In 2021, these viruses were detected in North America and later in South and Central America in 2022, infecting multiple animal species, including wild birds, poultry, wild mammals, humans, and, more recently, livestock [9,10].

Increased outbreaks of HPAI A(H5N1) in mammals have raised concerns about the virus’s ability, in such cases, to adapt to and infect humans more easily compared to birds, as non-human mammals share more biological features with humans and may act as mixing vessels for avian influenza viruses, resulting in the emergence of novel strains more virulent to animals and humans [6]. As a result, HPAI A(H5) viruses pose a devastating risk to animal and human health, affecting farmers’ livelihoods, international food trade, and threatening food security and public health [11].

## 2. IAV Genome Composition

**Figure 1:**
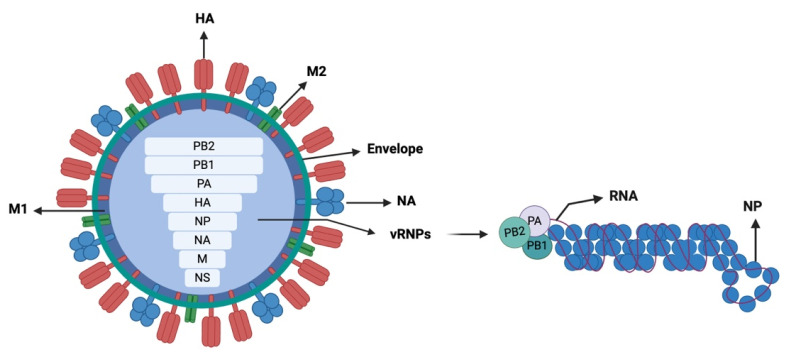
Diagrammatic representation of the influenza A virus particle, showing the HA trimer and NA and M2 tetramer proteins embedded into the host-derived lipid envelope. The M1 protein located beneath the viral envelope. All eight RNA segments are encapsulated with the viral envelope, with each segment bound by polymerase complex and coated by nucleoprotein, forming the viral ribonucleoprotein (vRNP) (shown on the right). The eight vRNA segments are arranged from top to bottom according to their sequence lengths, with PB2 the longest. Diagram created in https://BioRender.com (accessed on 2 February 2025).

Commonly referred to as influenza A virus (IAV), IAVs are a member of the genus Alphainfluenzavirus within the family Orthomyxoviridae [10,12,13]. These viruses are characterized as small, pleomorphic particles, initially measuring 80–120 nm in diameter, which gradually adopt a more spherical morphology [13,14]. As depicted in Figure 1, structurally, the viral particles consist of three key components: a host-derived lipid bilayer envelope that incorporates the HA and NA glycoproteins alongside the M2 protein; an inner shell formed by the matrix (M1) protein, providing structural integrity; and the nucleocapsids at the core, composed of multiple nucleoprotein (NP) molecules encapsidating the viral RNA [13,15].

Segments 1 to 3 of the IAV genome encode the RNA polymerases PB2, PB1, and PA, which are responsible for RNA transcription and replication [16]. Segment 5 encodes viral NP, which plays an important role in the assembly of the viral RNA. Segment 7 encodes M protein, which consists of two key proteins: the matrix protein (M1), which forms a layer beneath the viral lipid envelope and helps with nuclear export of the viral ribonucleoprotein (vRNP), and the ion channel protein (M2), which is embedded within the viral envelope and helps in the fusion of the viral and endosomal membranes [15]. Segment 8 encodes NS, which is responsible for evading the host innate immune response (NS1) and the nuclear export of the vRNP (NEP/NS2) [15,17].

Finally, and importantly, segments 4 and 6 of the IAV genome encode for HA and NA proteins, respectively, which play critical roles in the viral life cycle. HA facilitates the virus’s attachment to sialic acid receptors on the host cell surface, initiating viral entry into the cell and fusion of the viral envelope with the host endosomal membrane following endocytosis, enabling the release of viral RNA into the host cell cytoplasm [15,16]. NA, on the other hand, is essential for the release of newly assembled virions. It cleaves sialic acid residues on the host cell surface, preventing the virions from clumping together or reattachment to the host cell, facilitating the efficient release of progeny viruses to spread and infect new host cells [17].

Continued evolution is crucial for IAVs to cause seasonal outbreaks, annual epidemics, or occasional pandemics [13], with genetic reassortment, i.e., exchange of viral gene segments, being the hallmark of the virus in this process [18]. Reassortment occurs with each of the RNA gene segments, although it is most prevalent with the surface glycoproteins, likely due to immune pressure [13,18,19]. Different mechanisms, such as antigenic drift, antigenic shift, defective-interfering particles (DIPs), and RNA recombination, accumulate changes in the genomic segments of influenza viruses, leading to the evolution and variation of the organism [13]. Nonetheless, there are host-specific restriction factors that establish barriers to virus adaptation and evolution.

## 3. IAV Infectious Cycle: The Host and Virus-Specific Determinants of Replication

**Figure 2:**
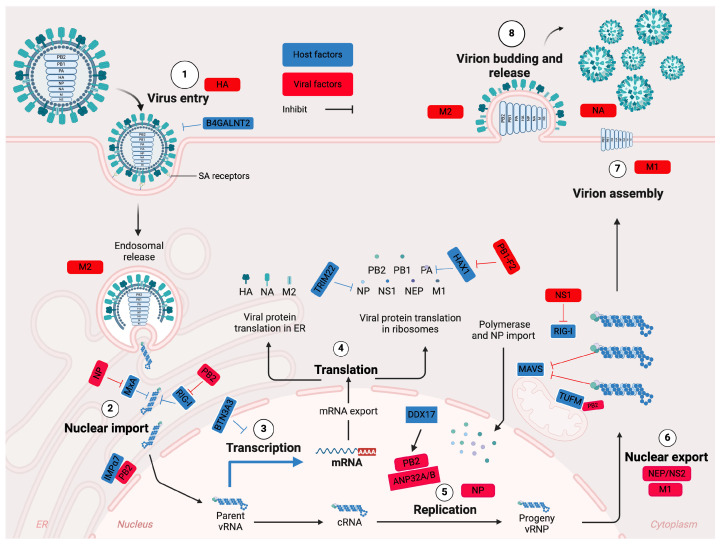
Infectious cycle of influenza A virus, with the host and virus-specific determinants in influenza virus genome replication, created in https://BioRender.com (accessed on 2 February 2025).

The infectious cycle of IAV (Figure 2), along with the host and virus-specific factors that influence influenza virus replication, is comprehensively reviewed elsewhere [17,20,21]. Figure 2 summarizes the key points.

HA molecules on the virus envelope initiate the influenza A virus infectious cycle by attaching the virus to the host cell’s sialic acid (SA) receptors (Step 1). The glycosyltransferase B4GALNT2, modifying glycans with α2,3-linked sialic acids (SAs), can prevent AIV entry. The virion enters the cell by endocytosis, where the acidic environment of the endosome activates the M2 ion channel and causes permanent structural changes in the HA, exposing the hydrophobic fusion peptide and triggering viral envelop fusion with the endosomal membrane. Acidification of the virion interior, mediated by the influx of potassium ions and protons through the M2 ion channel, dissociates M1 from vRNP, allowing the release of vRNP into the cytoplasm. Here, retinoic acid-inducible gene I protein (RIG-I), which is a “cytoplasmic pattern recognition receptor” responsible for detecting RNA molecules during replication, and MxA (“interferon-regulated resistance GTP-binding protein” also known as Mx1) sequester the vRNPs within the cytoplasm. However, mutations in NP (i.e., 313Y/V and 52N/H/Q) and in position 627 PB2 (e.g., 627K) enable the mammalian-adapted vRNP to evade binding by MxA and escape detecting by RIG-I, respectively. The mammalian adaptation mutations in PB2 enable the use of importin-α7 (IMPα7), which is a key nuclear transporter protein, thereby enhancing the nuclear entry of the vRNPs. The vRNPs are transported to the nucleus through the nuclear pore complex (Step 2).

Inside the nucleus, the vRNPs serve as templates for primary transcription, producing viral mRNA (Step 3). Here, BTN3A3 (butyrophilin subfamily 3 member A3), which is an interferon-induced restriction factor, blocks transcription of avian IAVs. However, NP mutations (i.e., 313Y/V and 52N/H/Q) can escape this barrier. The synthesized mRNA is transported to the cytoplasm, where translation occurs using host cellular machinery (Step 4). Translation occurs in two distinct locations: HA, NA, and M2 are synthesized on endoplasmic reticulum (ER)-associated ribosomes, while PB2, PB1, PA, NP, NS1, NEP, and M1 are synthesized on free ribosomes in the cytoplasm. Tripartite motif-containing protein 22 (TRIM22) can degrade the mammalian-adapted NP, while HS1-associating protein X1 (HAX1) can inhibit PA, which can be antagonized by PB1-F2. Replication and secondary transcription of the vRNPs are initiated when the newly synthesized NP and polymerase complex are transported back into the nucleus (Step 5). Viral RNA (vRNA) replication from the complementary RNA (cRNA) is promoted by the acid leucine-rich nuclear phosphoprotein 32 family member A or B (ANP32A/B) and small viral RNAs (svRNAs). IAV polymerase must acquire mammalian-adaptation mutations (e.g., E627K PB2) to use human ANP32A/B. The DEAD-box protein 17 (DDX17) enhances the activity of PB2 627K and restricts the activity of PB2 627E. NEP and M1 then export the newly synthesized vRNPs from the nucleus to the cytoplasm (Step 6).

Here, numerous cell sensors identify viral particles, including the vRNPs. AIV PB2 627E is bound by mitochondrial Tu elongation factor (TUFM), increasing autophagy and suppressing viral replication, whereas the mammalian adapted PB2 (627K), as well as PB1-F2 anchors to mitochondria, where they evade recognition by mitochondrial antiviral signaling protein (MAVS). NS1 interacts with RIG-I and inhibits its activation, preventing the downstream expression of interferon-stimulated genes (ISGs) and associated antiviral effects.

HA exists in an immature form known as HA0, which lacks the ability to induce membrane fusion. Therefore, in the Golgi apparatus, HA is cleaved into HA1 and HA2, which can occur either at a single basic amino acid site (monobasic) or at a sequence of multiple basic amino acid sites (multibasic). While the monobasic cleavage site is associated with human IAVs and low pathogenic avian influenza viruses (LPAI), the multibasic cleavage sites are typically associated with highly pathogenic avian influenza viruses (HPAI) H5 and H7 subtypes and are recognized and cleaved by Furin, which is ubiquitously expressed. The vRNPs are then delivered to the host cell surface for packaging, which is facilitated by M1, where they are assembled with the viral structure proteins, including HA, NA, M1, and M2 (Step 7).

Newly assembled virions, encompassing internal and structural proteins, bud off the host cell plasma membrane, with their release facilitated by membrane scission carried out by M2 (Step 8). NA facilitates the subsequent spread of the virions to neighboring host cells by cleaving sialic acid residues from the cell surface, viral particles, and mucus. Avian IAVs, which have short-stalk NAs, cannot bypass the human respiratory tract mucus, whereas the long-stalk NAs of the mammalian-adapted viruses allow them to overcome this barrier.

## 4. IAV Subtype Diversity and Classification

Based on the antigenic properties of the IAVs’ HA and NA surface glycoproteins [22], currently, there are 19 known HA and 11 NA subtypes [23]. In birds, 17 HA and 9 NA subtypes of IAVs have been identified to date [23], exhibiting a variety of HA/NA combinations. Influenza A appears to be fully adapted to aquatic wild birds and causes few or no clinical signs in these hosts [13]. Birds, with variations among different species, can be infected with all the currently known subtypes of IAVs, except subtypes A(H17N10) and A(H18N11), which have only been isolated from bats and are poorly adapted to non-bat species [24,25,26]. The virus preferentially infects the intestinal epithelial cells of ducks and is secreted in abundance in their feces [13,22,27,28]. It remains infectious in fecal materials at 4 °C for 30 days and at 20 °C for at least 7 days [28]. The virus’s extended survival and infectiousness in the environment facilitate its transmission both within and between species [27].

The behavior of IAVs in gallinaceous birds has distinguished these viruses into two categories: highly pathogenic avian influenza (HPAI) and low pathogenic avian influenza (LPAI), based on the mortality rate of infected chickens [29,30]. Most known IAV strains exhibit low pathogenicity and are, thus, carried with little or no clinical manifestations [31]. LPAI viruses generally cause asymptomatic or mild respiratory signs in poultry and wild birds; however, some LPAI viral strains, e.g., H7N9, have caused life-threatening infections and death in humans [1]. Insertion of basic amino acid residues in the HA0 cleavage site can shift the LPAI phenotype to HPAI [32]. HPAI viruses, on the other hand, can cause severe damage to tissues and vital organs in poultry, resulting in the rapid onset of clinical symptoms ranging from respiratory distress to neurologic signs, often progressing to systemic failure followed by 100% mortality within 36–48 h post-infection [1,12,33,34]. HPAI can also lead to significant morbidity and mortality in wild birds, further highlighting its devastating impact on avian populations [31].

Although HPAI viruses responsible for systemic infections and significant mortality in poultry are mainly associated with the H5 and H7 subtypes [33], not all strains of these subtypes are highly pathogenic [12]. The pathogenicity and virulence of HPAI vary significantly across different virus strains and avian species, with some strains causing more severe clinical signs than others in certain bird species [35]. Infected birds, whether symptomatic or not, can shed high quantities of the virus in the environment, furthering transmission and evolution of the virus [35,36].

## 5. Evolution and Global Distribution of HPAI A(H5N1) Clade 2.3.4.4b

Influenza viruses were initially isolated from chickens in 1902 (A/Brescia/1902/H7N7), from swine in late 1920, from humans in early 1930, from domestic ducks and horses in the 1950s, from terns in 1961, and since 1974, from different species of shorebird waterfowl [13].

Wild aquatic birds, especially those of the orders Anseriformes (waterfowl, including geese, ducks, and swans) and Charadriiformes (shorebirds and gulls), are well-established reservoirs and natural hosts for most IAVs [1,13,22,36,37,38,39,40,41,42,43,44,45,46,47]. These birds play a critical role in perpetuating numerous subtypes of avian influenza viruses (AIVs) through asymptomatic viral shedding [48]. The transmission cycle involves immunologically naïve immature birds becoming infected, which subsequently facilitates viral spread to other susceptible individuals [49]. Most of these viruses exhibit low pathogenicity in their natural avian hosts and have been isolated from more than 25 families and approximately over 100 wild bird species, highlighting the broad distribution of AIVs among free-living aquatic bird populations [38,43]. While asymptomatic in their natural hosts, these low pathogenic viruses have given rise to highly pathogenic avian influenza (HPAI) strains [12,50,51], leading to life-threatening infections in humans and various other animal species [1].

Highly pathogenic avian influenza viruses of the H5 subtype, HPAI A(H5), are the most widely detected subtype of the HPAI viruses and are responsible for numerous outbreaks in the host animals, particularly Gallinaceous birds, around the world [8]. HPAI A(H5) remains a global threat to poultry and wild birds, as well as a significant concern for food security and public health due to their high pathogenic potential [3,52,53]. HPAI A(H5) was initially isolated from shorebirds in 1961 (A/Tern/South Africa/61/H5N3) and subsequently from turkeys in Canada in 1966, chickens in Pennsylvania in 1983/84, turkeys in England in 1991/92, chickens in Mexico in 1994/95, and geese in China in 1996 (A/Goose/Guangdong/1/96 (H5N1)) [54].

HPAI, as a global crisis, is primarily associated with the A/goose/Guangdong/1/96 A(H5) (H5 gs/GD) lineage [55]. Due to the accumulation of mutations and reassortment with other subtypes of influenza viruses, the A(H5) gs/GD lineage has evolved into ten clades, designated as clades 0–9, along with multiple subclades based on the HA gene [3,8,48,56]. While the viruses of this lineage were initially confined to Southeast Asia during the first five years following their detection, they crossed the species barrier, infecting poultry, wild birds, and humans, and spread via migratory birds to Europe, Africa, and North America [3,57,58,59]. Demonstrating an unprecedented potential for reassortment [60], HPAI viruses of clade 2.3.4.4 of H5Nx have caused extensive outbreaks worldwide, evolving into eight additional subclades, i.e., 2.3.4.4a–2.3.4.4h, [48]. Owing to the extensive bird migrations, some of these subclades have undergone significant reassortments with LPAI viruses in wild birds across migratory flyways [10].

## 6. Wild Bird Migration Patterns and Spread of AIVs

Historically, migratory birds have been linked to the emergence and spread of influenza viruses, including pandemic strains, as well as the HPAI A(H5N1), in humans and animals [1,4,10,13,45,59,61,62]. Migration, ranging from local to intercontinental movements, is common in birds residing in seasonal habitats [38]. These movements connect wild birds from across different countries/continents together while aggregating at breeding or wintering sites across the large flyways, facilitating the spread and spillover of the virus [38,63] and, meanwhile, challenging virus surveillance and control measures [62]. The overlap of the birds within the seasonal habitats can readily facilitate the spread of AIVs from the infected birds to the susceptible contacts [4,50,64].

Migratory birds generally move north–south along routes between nesting and wintering sites, with higher densities of birds following natural landscapes such as coasts, mountains, and river valleys [64,65]. These routes, known as migratory flyways, provide essential guidance for their seasonal movements. The North American migratory flyway consists of four major flyways, i.e., Pacific, Central, Mississippi, and Atlantic (https://tpwd.texas.gov/huntwild/wild/birding/migration/faq/#a16 (accessed on 21 February 2025). Except for the coastal regions, these flyways overlap in some areas in northern breeding and southern wintering sites, exhibiting less clear migratory boundaries [65], which results in the mixing of the residential birds with the migrant birds from a range of species that aggregate at the wintering, breeding, or stopover sites [64], fostering the spread of the virus within these avian populations [38,63,64].

In addition to the geography and direction of the movement, the timing of migration is another determinant of AIV spread and prevalence among different wild bird species [27,64]. Prevalence of AIVs in wild ducks in North America was estimated to be as high as 22.2% during their southern migration in the fall, coinciding with the birth of a large number of immunologically naïve wild birds [64,66] that are more susceptible to the infection [49,67,68]. Likewise, in the northern hemisphere, peak AIV prevalence occurs in August and September, when juvenile birds contract the infection as they gather in Canadian marshaling sites before migration, with up to 30% of the birds shedding the virus [13]. However, when the birds reach the lower Mississippi, the AIV prevalence gradually declines [27] to 1.6–2% in November and to 0.4% in December and January when they reach Louisiana [13].

Migration can also play an important role in the introduction of novel AIV strains into a new habitat [64]. A study between 1998 and 2000 in Minnesota, U.S., detected a higher prevalence of previously uncommon AIV subtypes, including H5, H7, and H9, from wild bird samples collected annually during mid-September, which is a period coinciding with the arrival of new migratory birds [68]. Finally, migration and migratory flyway boundaries can play an important role in shaping the genetic structure of AIVs, as evidenced by the phylogenetic separation of AIV genotypes into distinct Eurasian and American lineages [40,42,69].

Many wild birds, particularly those from Charadriiform and Anseriform species, typically move long distances, acting as hidden carriers favoring the transmission of LPAI viruses within countries and even continents [1,38]. These movements have been recognized as the critical drivers for the persistence and broad geographic distribution of avian influenza viruses [10,70].

## 7. Bird Species Favoring Persistence and Transmission of AIVs

Wild birds, including waterfowl, gulls, and shorebirds, constitute the natural reservoirs of IAVs. Throughout their annual migration cycles, these birds engage in long-distance migrations that can facilitate the intercontinental movement of AIVs [37,71]. These movements can also impact the subtype diversity of AIVs due to variations in host immunity [1] or the commingling of different bird populations at stopovers or wintering sites [71].

Geographical separations between host species distinguished AIVs into two phylogenetically distinct superfamilies: the American lineage (New World) and Eurasian lineage (Old World) [34,40,42,69]. However, the presence of gene segment(s) from one superfamily in viruses of the other [19,42] has blurred the geographical boundaries between these lineages. For example, Eurasian lineage LPAI viruses of the H6 subtype were detected in North American Mallard Ducks, with a subsequent spillover into poultry, causing an AIV outbreak in California between 2000 and 2002 [72]. The Eurasian lineage HPAI A(H5) 2.3.4.4c, in 2014–2015, and the Eurasian lineage HAPI A(H5) 2.3.4.4b were detected in multiple animal species, including humans, in North America [10,73], with Mallard Ducks (*Anas platyrhynchos*) and ruddy turnstones (*Arenaria interpres*) postulated to be the potential donors [10].

### 7.1. Waterfowl

Waterfowl species, particularly dabbling ducks, have frequently been the target of AIV surveillance [27,67,74,75,76], likely due to their behavior, distribution, availability, and accessibility, making them ideal subjects for AIV surveillance efforts. In a multidecade surveillance of AIV in wild waterfowl performed between 1976 and 2015 throughout the central and Mississippi migratory flyways, dabbling ducks accounted for 91% (*n* = 70,704) of the total samples collected [67]. Remarkably, dabbling ducks have also represented 98% of the total IAV isolates detected in this study, representing a vast IAV subtype diversity, including H1-H12, H14, and N1-N9 [67]. In a multi-year study from 1998 through 2006, screening 36,809 samples from 323 bird species of 18 orders, dabbling ducks comprised the highest prevalence of AIVs among all, harboring almost all HA subtypes, except H13 and H16—which were primarily detected in gulls [27]. Additionally, in the current outbreak of the HPAI A(H5N1) clade 2.3.4.4b in the U.S., a higher prevalence of these viruses has been detected among dabbling ducks, notably in Mallard Ducks (15.48%), compared to other wild bird species [73], thereby underscoring the role these birds play in the persistence and transmission of AIVs. The avirulent nature of AIVs in ducks, combined with the abundant opportunity for virus transmission within and between species during the yearly breeding and wintering congregations, creates an optimal scenario for ongoing AIV circulation within this population [13,49], further emphasizing the pivotal role of ducks in the perpetuation of AIVs.

Several factors may contribute to the higher prevalence of AIVs in dabbling ducks. First, the increased prevalence of AIVs in dabbling ducks could be attributed to their behavior, namely, their preference for shallow-water habitats, where they shed the virus into surface water, facilitating fecal–oral transmission to susceptible bird populations [27,67]. Second, the higher detection of AIVs in dabbling ducks may be due to their population structure, which includes a larger proportion of immunologically naïve ducks. Studies show that the annual mortality rate of adult mallards is approximately 40% [27], leaving a greater proportion of the population composed of juvenile ducks that are immunologically naïve and, therefore, more susceptible to AIVs [13,77]. A third factor contributing to the elevated frequency of AIVs in dabbling ducks could be their overrepresentation in surveillance efforts compared to other bird species [67,75]. Nevertheless, the higher prevalence of AIVs in these wild bird species highlights their role in the persistence and perpetuation of AIVs [49] and enhances our understanding of the virus’s evolution and ecology.

In contrast to dabbling ducks, diving ducks exhibit different behaviors and habitat preferences. They often use marine habitats and forage deep beneath the surface of the water, with some species, such as geese and swans, grazing in agricultural lands and pastures [27]. These ecological behaviors lead to less efficient AIV transmission dynamics and, therefore, a lower prevalence of the virus in these bird species [27]. Nevertheless, the role of diving ducks has been found to be critical in AIV evolution and persistence. For instance, low-pathogenic avian influenza (LPAI) viruses in diving ducks likely contributed to the emergence of novel HPAI A(H7N8) viruses [51]. Additionally, a unique IAV hemagglutinin (HA) gene segment, designated as H19, was recovered from two Lesser Scaup Ducks (diving ducks) in northern California [78]. These findings underscore the importance of diving ducks as a target for AIV surveillance

Additionally, sea ducks have been identified as key players in the persistence and spread of IAVs. For example, the H14 subtype of influenza A virus, harboring an unidentified NS segment, was isolated from sea ducks across the North American Mississippi migratory flyway for the first time since its initial detection in 1982 [76,79]. The undetected Eurasian lineage H10 subtype was isolated from sea ducks for the first time in North America [80]. Furthermore, sea duck-origin IAV harboring a highly divergent, unique H4 subtype was detected in North America [75].

Accordingly, in addition to dabbling ducks, efforts should be made to include often underrepresented wild waterfowl species, such as sea ducks and diving ducks, in AIV surveillance programs [75].

### 7.2. Gulls and Shorebirds

In gulls and shorebirds (Charadriiformes), behavioral factors such as aggregation at breeding and wintering sites, colony breeding, feeding patterns, and mixing with other wild bird species likely influence AIV ecology and prevalence [27]. These birds often migrate between continents and aggregate in large groups at both breeding and non-breeding sites, providing opportunities for long-distance, intercontinental movement of AIVs [37,42,74]. Additionally, when comparing subtype diversity among different aquatic bird species, studies have found a higher frequency of outsider gene segments—those isolated in either the Americas or Eurasia but belonging to the opposite lineage—in gulls and shorebirds than in ducks [42]. This suggests their important role in the intercontinental movement of AIV gene segments. Furthermore, the H16 subtype of AIVs has only been isolated from gulls and shorebirds, not from ducks [42], indicating these birds carry unique AIVs.

A comprehensive, multi-year study conducted over 16 years, from 1985 to 2000, tested 4266 cloacal swab samples collected from Canadian shorebirds and gulls [66]. The findings revealed that 14.2% of the samples tested positive for AIVs. This study also found that shorebirds and gulls exhibited greater hemagglutinin (HA) subtype diversity compared to ducks, with a much higher prevalence of AIVs in shorebirds (14.2%) compared to wild ducks (0.03–0.3%) during northern migration in spring. Therefore, shorebirds and gulls are important players in the intercontinental spread of AIV genes [42,81].

While most of the AIV subtype diversity has been documented in ducks in a multi-year study in Europe, H13 and H16 subtype AIVs were primarily detected in gulls [13,27]. In contrast to waterfowl, which primarily carry only Eurasian-lineage AIV segments, detections of entirely Eurasian-lineage (EA), American-lineage (Am), and/or mixed EA/Am-lineage AIV segments in gulls in Iceland suggest that gulls may harbor a greater diversity of AIVs than waterfowl [37,42]. North American AIVs detected in gulls were also found to possess both EA and Am lineage AIV reassortant gene segments [74]. These findings suggest ecological variations in AIVs between waterfowl and gulls, highlighting the role of gulls as mixing vessels for different lineages of AIVs [74].

Therefore, a comprehensive AIV surveillance strategy should include both shorebirds and gulls (Charadriiformes), as well as waterfowl, to enable more effective monitoring of the evolution, distribution, and persistence of AIVs within these natural reservoirs.

## 8. Arrival of the HPAI A(H5) Clade 2.3.4.4b into the U.S.

The first intercontinental spread of the Eurasian A(H5N2/N8) clade 2.3.4.4c to North America occurred during the 2014–2015 outbreak, which affected 21 U.S. states and over 50 million poultry, including 42.1 million chickens and 7.5 million turkeys, with detections in wild waterfowl [10,33]. In 2016/2017, outbreaks of Eurasian A(H5) clade 2.3.4.4b H5N8 (in 2016) and H5N6 (in 2017) occurred in Asia and spread to Europe and Africa, leading to significant wild bird die-offs in 2016 and limited mortality in 2017 [82]. From December 2019 to June 2020, reassortment between the EA lineage LPAI and HPAI A(H5N8) viruses from Africa [55,83] led to outbreaks of clade 2.3.4.4b in Eastern and Central Europe [30,82]. A novel H5N8 strain from this clade was detected in mute swans in the Netherlands in October 2020 [84], spreading widely across Europe and causing extensive poultry and wild bird outbreaks [30,53,85,86]. This outbreak primarily impacted geese, including Gre Lag Goose (*Anser anser*) and Barnacle Goose (*Branta leucopsis*), as well as Swans, notably Mute Swans (*Cygnus olor*) [82], with viruses sharing a genetic similarity to clade 2.3.4.4b HPAI H5 viruses detected during 2018–2019 in Egypt [84].

Before the detection of clade 2.3.4.4b in Europe, genetically related HPAI A(H5N8) viruses were found in Iraq, southern central Russia, and Kazakhstan in May 2020, suggesting direct spread to Europe or transmission via migratory wild birds moving from Siberian breeding sites to European wintering grounds [30,87]. The 2016/2017 outbreaks in Europe mainly affected Eurasian Wigeons and Tufted Ducks in the Netherlands [41]. However, the 2020/2021 outbreak involved fewer duck infections, indicating differences in the pathogenicity of clade 2.3.4.4b among wild bird species over time [82]. From 2014 to 2020, HPAI A(H5) clade 2.3.4.4 viruses caused significant losses in the European and U.S. poultry industries [10,84].

The persistence of A(H5) clade 2.3.4.4b was limited to the Eurasian flyways until December 2021, when the first U.S. outbreak was detected through routine surveillance of wild ducks in South Carolina and North Carolina [9,10]. Prior to this, the virus had been found in seabirds and poultry in Canada [10]. The virus likely spread transatlantically via wild birds through the North Atlantic region, as evidenced by the high genetic similarity between the Canadian isolates and those detected in northwestern Europe in 2021 [88]. The introduction of EA-origin HPAI A(H5N1) into the U.S. was likely via the Atlantic flyway, with the virus dispersing from northern Europe, passing through Arctic regions, and moving southward into Canada and the U.S., facilitated by migratory wild birds [9].

From December 2021 to April 2022, three independent introductions of the HAPI A(H5N1) clade 2.3.4.4b were identified in the United States. Two of these introductions, designated as A1 and A2 genotypes, were detected in east coast states, while the third, recognized as the A3 genotype, was reported in Alaska in April 2022 [10]. A2 and A3 represent unreassorted genotypes, while EA lineage viruses of the A1 genotype are reassorted with North American low pathogenic avian influenza (NAm LPAI) viruses from wild birds, involving five internal gene segments, i.e., PB2, PB1, PA, NP, and NS [10].

## 9. Virus Incursion and Spread Behavior: An Emerging Risk to the United States

In this section, we will review the key drivers behind the spread of HPAI A(H5N1) clade 2.3.4.4b within and between species, emphasizing critical events and associated complications.

### 9.1. Within Wild Birds

Wild birds, when infected with AIVs, can expel a high concentration of the virus in their feces, mucous, and saliva [89], contaminating the environment, which facilitates transmission to other susceptible birds. In aquatic birds, surface water acts as a significant conduit for the virus, with its survival depending on factors such as water pH, salinity, and temperature, allowing the virus to persist for extended periods. Birds that dabble or preen in contaminated water are at risk of infection [27,63,90]. The primary transmission route includes fecal–oral transmission, as well as preening, allopreening, and cloacal drinking, which can spread the virus among susceptible contact birds [90]. Sharing a common water resource further promotes inter- and intra-species spread of the AIVs among bird populations [64].

The first cases of the EA lineage HPAI A(H5N1) clade 2.3.4.4b in U.S. wild birds detected on January 12 and 13, 2022, among Gadwall, Northern Shoveler, Blue-Winged Teal, and American Wigeon, all sampled on December 30, 2021, in North and South Carolina through surveillance efforts conducted among hunter-harvest waterfowl [73]. Shortly after these initial detections, the virus spread among aquatic birds, following bird migratory patterns and circulating across various flyways. As of Nov 20, 2024, HPAI A(H5N1) viruses have been detected in 202 wild bird species, representing 10,604 individual birds across all 50 U.S. states and all four migratory flyways [73]. The highest detections of the virus have been reported in Minnesota (6.43%; n = 682), which is part of the Mississippi migratory flyway, while the lowest detections were reported in Washington DC (0.03%; n = 3) and West Virginia (0.03%; n = 3), both of which belongs to the Atlantic flyway [73,91].

In this outbreak, compared to the EA/NAm reassorted viruses, non-reassortant EA lineage HPAI A(H5) strains represent the highest prevalence of the virus, accounting for 63% of the total detections documented in wild birds, as of Nov 20, 2024 [73].

**Figure 3:**
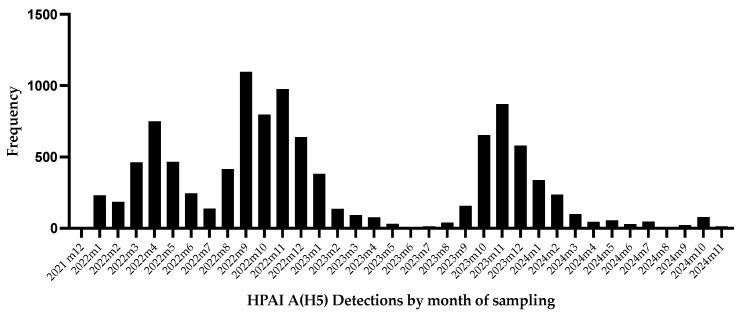
Epidemic curve of HPAI A(H5) in wild birds in the U.S. since start of the outbreak in December 2021 through November 2024. Dataset obtained from the USDA [73] accessible at https://www.aphis.usda.gov/livestock-poultry-disease/avian/avian-influenza/hpai-detections (accessed on 29 November 2024).

Furthermore, exploring the epidemic curve of the HPAI A(H5) detections in wild birds in the U.S. (Figure 3), the epidemic seems to be a common source point event with secondary transmission. After the initial detections in North and South Carolina, the virus was widespread across all the U.S. migratory flyways, facilitated by widespread transmission both within and between species.

**Figure 4:**
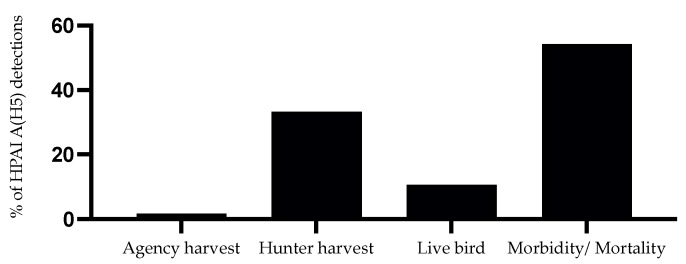
Surveillance methods contributed toward the detection of HPAI A(H5) clade 2.3.4.4b in the U.S. wild birds from January 2022 through November 2024. Data are obtained from the USDA [73] accessible at https://www.aphis.usda.gov/livestock-poultry-disease/avian/avian-influenza/hpai-detections (accessed on 29 November 2024).

Lastly, regarding HPAI A(H5N1) outbreaks in wild birds in the U.S., it is important to recognize the role of surveillance methods in facilitating these detections. As shown in Figure 4, morbidity/mortality sampling followed by hunter–harvest surveillance efforts were the primary contributors to these detections [73], highlighting the role of these methods in the surveillance of influenza A viruses and, thereby, our understanding of the virus evolution and spread.

### 9.2. Birds and Poultry

Domestic birds, such as poultry, can contract AIVs from wild birds through direct contact with infected birds or indirect contact with contaminated surfaces, such as water, bedding, food, vehicles, boots, etc., which may be contaminated with wild bird feces [59]. The abundant resources on farms—water, food, and shelter—can attract wild birds [92], creating an important pathway for the transmission of AIVs from wild birds to poultry. Outbreaks of HPAI A(H5N1) clade 2.3.4.4b in U.S. poultry farms are primarily linked to point-source introduction of the virus from wild migratory birds, followed by limited farm–farm transmission [10]. The likely pathway for these incursions is the access of infected wild birds to the poultry premises. From 8 February 2022 through 20 November 2024, HAPI A(H5) clade 2.3.4.4b outbreaks in the U.S. have affected 110.47 million poultry across 1231 flocks across 49 states [73].

AIVs are circulating in wild birds and pose a significant threat to poultry and human health. Therefore, it is essential to intensify biosecurity measures and strengthen surveillance and monitoring programs to safeguard animal and public health [1,55].

### 9.3. Birds and Wild Mammals

HPAI Infection in wild mammals can lead to severe morbidity and mortality [73]. The first report of sporadic spillover of HPAI A(H5N1) belonging to clade 2.3.4.4b from wild birds into wild mammals was on 1 April 2024, when reports of sick or dead wild mammals were submitted to the state wildlife agencies [93,94,95,96]. Since then, the virus has been detected in multiple wild mammal species across the United States. The wild mammal species affected are primarily those that hunt and/or feed on wild birds [95]. Among these species, the red fox (*Vulpes vulpes*) has the most frequently reported incidence of HPAI H5N1, likely due to its broad distribution, which overlaps with the geographical range of the HPAI A(H5N1) outbreaks and/or its dietary habits of hunting and feeding on wild birds [94,95]. Since wild waterfowl and many other wild birds are common or incidental natural dietary components of wild mammals [94], the ingestion of infected wild birds may be the primary mode of HPAI transmission into wild mammals [70,94,95].

As a consequence of the HPAI A(H5N1) clade 2.3.4.4b outbreaks in wild mammals, several wild mammal species have experienced significant mortality events. Notable cases include harbor seals (*Phoca vitulina*) along the Maine coast, U.S., during the summer of 2022; American minks (*Neovison vison)* in Spain in October 2022; and sea lions (*Otaria flavescens*) in Peru between January and April 2023 [89]. The harbor seal HPAI A(H5N1) outbreak in the U.S. coincided with an HPAI outbreak in symptomatic wild birds in the region [70]. The transmission route of the virus to seals is thought to involve the environmental shedding of the virus from wild birds. However, data do not support the seal-to-seal transmission of the HPAI A(H5N1) clade 2.3.4.4b [70]. Additionally, while most seals were found dead, respiratory and neurological signs were documented in the few that survived [95].

The HPAI A(H5N1) outbreak in American minks in Spain coincided with an outbreak in seabirds, suggesting that wild birds were the likely source of HPAI A(H5N1) introduction to the farmed minks, followed by mink-to-mink transmission of the virus [97]. However, the exact source of this outbreak remains unknown [97]. Infected mink exhibited clinical signs of loss of appetite, depression, bloody snouts, hypersalivation, and neurological signs, including tremors and ataxia [97]. The HPAI A(H5N1) outbreak among sea lions in Peru, which resulted in 5224 deaths, was likely introduced by infected wild birds cohabiting on the Peruvian coastline with the sea lions, either through direct contact with infected live birds or by scavenging their carcasses [98]. However, no sea lion-to-sea lion transmission of the virus was documented [98].

**Figure 5:**
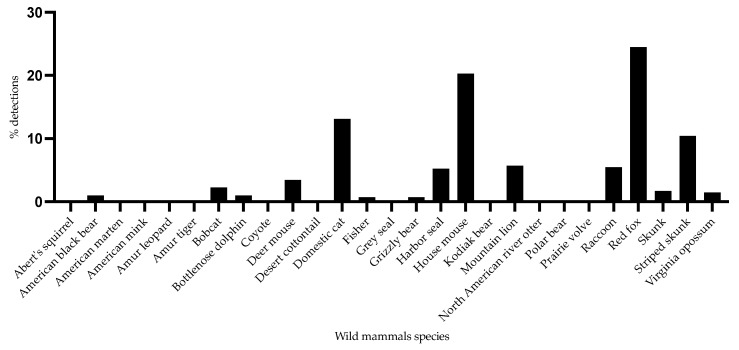
HPAI A(H5N1) clade 2.3.4.4b detections in the wild birds in the U.S. Dataset, obtained from the USDA [73], accessible at https://www.aphis.usda.gov/livestock-poultry-disease/avian/avian-influenza/hpai-detections (accessed on 20 November 2024).

In the U.S., as of 20 November 2024, outbreaks of HPAI H5N1 clade 2.3.4.4b were detected among 27 wild mammal species, representing 404 individual wild mammals, detected across 32 states since its initial detection on 5 May 2022 [73]. Among the wild mammal species, red fox (*Vulpes vulpes Linn*), followed by striped skunk (*Mephitis mephitis*), represents the highest frequency of HPAI detections, 79 and 32 detections, respectively (Figure 5) [73].

Compared to the reassorted EA/AM-lineage H5N1, EA lineage H5N1 accounts for the highest frequency of detections in wild mammals in the U.S., accounting for 68% of detections [73].

Infection with HPAI H5N1 viruses has been reported in multiple mammal species [99,100,101,102], scavenging birds, and raptors [88,103,104], typically due to the ingestion of infected wild birds. Yet, evidence of horizontal transmission of HAPI H5N1 in wild mammals has not been firmly established [94].

In Canada, H5N1 viruses of clade 2.3.4.4b caused sporadic infections in 40 mesocarnivore species (including American mink, red foxes, and skunks). Seventeen percent of these infections harbored mammalian adaptive mutations in the PB2 segment, potentially facilitating viral replication, interspecies transmission, and the threat of human infection [93]. The continued spread and persistence of HPAI viruses in mammals may lead to further reassortment or adaption of the virus to a broader range of mammalian hosts, underscoring the need for continued surveillance and monitoring of HPAI viruses in these populations to better understand evolution, spread, and spillover of the virus [94].

The colonial nature of wild birds and wild mammals may facilitate enhanced interspecies transmission and circulation of influenza viruses, leading to antigenic shifts and subsequent widespread distribution of the virus over broad geographic regions through migratory wild birds [70]. This highlights the necessity of a One Health approach in surveillance and monitoring at the wildlife interface [70,101] in order to better understand the evolution of the virus and its emerging risk to humans.

### 9.4. Birds and Livestock

The central flyway is one of the major migratory flyways in North America, spanning from the Arctic regions of Canada and Alaska in the north, through the central United States, and down to Central and South America, including Mexico [91]. Texas, which is located within the central flyway, also often overlaps with the Mississippi migratory flyway during bird migrations, facilitating a wide spread of HPAI A(H5N1) viruses between migratory flyways and their associated habitats, including critical landscapes such as cattle farms [105].

Anorexia, decreased milk production, yellow and thickened milk resembling colostrum, and other flu-like symptoms in lactating dairy cattle, along with reports of deceased wild birds and domestic cats on dairy farms in Texas, Kansas, and New Mexico, prompted investigations by the U.S. Department of Agriculture (USDA), Food and Drug Administration (FDA), Centers for Disease Control and Prevention (CDC), and state veterinary and public health officials. [106,107,108]. The illness primarily affected older cows in mid to late lactation, with symptoms peaking 4–6 days after onset and resolving within 10–14 days [106,107]. On 25 March 2024, rRT-PCR detection of EA lineage HPAI A(H5N1) clade 2.3.4.4b in milk samples from sick dairy cows, as well as in lung and brain tissue samples from domestic cats known to have consumed raw milk and colostrum on a Texas dairy farm, was confirmed by the National Veterinary Services Laboratories (NVSL) for the first time in one dairy herd in Texas [92].

Whole genome sequencing confirmed that all samples tested positive for HAPI A(H5N1) [107]. Several days later, the NVSL identified genome sequences of 11 additional HPAI A(H5N1) clade 2.3.4.4b viruses from six wild birds, four additional dairy cattle, and one skunk from Texas [107]. Genotypic characterization of these viruses revealed that all shared nearly 100% sequence homology, forming a new genotype within the HA clade 2.3.4.4b, designated as B3.13 [107]. B3.13 emerged due to reassortment, carrying PB2, PB1, PA, HA, NA, M, and NS genes from the B3.7 genotype, which emerged in 2023, and harboring the NP gene from an LPAI related to A/mallard/Alberta/567/2021 (H11N9) [107]. Time-scaled maximum clade credibility (MCC) phylogenetic analysis postulated this new genotype to be a descendant of viruses of wild bird origin [107], possibly spreading to the dairy farm in Texas via migratory wild birds [107,108], likely through the consumption of feed contaminated with the feces of infected wild birds [106].

Analysis of critical amino acid substitution and the tracking of mammalian adaptation mutations and antiviral resistance markers of all the HPAI A(H5N1) clade 2.3.4.4b isolates recovered from the affected dairy cows and two cats revealed significant mutations in HA (137A, 158N, and 160A). These changes are known to promote receptor-binding affinity of the virus to human-type receptors in M1 (30D, 43M, and 215A) and in NS1 (42S, 103F, and 106M), both of which are known to increase virulence [107]. However, critical mutations in PB2 (i.e., 271A, 292V, 591K, 627K/V/A, 701N), which could promote mammalian adaptation and virulence of the virus, as well as markers representing antiviral resistance, were absent in these sequences [107].

A dairy herd in Michigan, which had recently received cows from Texas, was also confirmed to have HPAI, with a strain similar to that confirmed in Texas and Kansas. This suggests possible horizontal transfer of the virus between cattle, likely through shared equipment and/or animal movement [73]. As of 20 November 2024, there have been 616 confirmed cases of HPAI A(H5N1) clade 2.3.4.4b in dairy cattle across 15 states [92]. The presence of the virus on dairy cattle premises resulted in clinical manifestations in the affected cows, including a notable drop in milk production and significant virus shedding in milk, and a high mortality (~50%) among the cats on the premises that consumed raw milk or colostrum from affected cows [106]. Infected lactating cows shed the virus abundantly in their milk (10^4^ to 10^8^ 50% tissue culture infectious dose, TCID50) [109], which could facilitate the spread of the virus to other susceptible mammals, including humans, through unpasteurized milk [106]. HPAI A(H5N1) clade 2.3.4.4b viral RNA was detected in the pasteurized milk by qPCR [110]. However, studies on the isolation of the viable virus from retail milk did not find viable viruses among the qPCR-positive milk samples, indicating the effectiveness of pasteurization in the inactivation of AIVs [111].

HPAI A(H5N1) clade 2.3.4.4b has established a broad host range with successful intra and inter-species transmission. These viruses have spread from wild birds to poultry, wild birds to livestock, poultry to human, cow to cow within and between farms (with the latter likely due to the cattle movement [112]), cow to human, cow to cats, and from cow to poultry [113,114]. Additionally, the virus has been detected in neonatal goats that were farmed in a single premise with cattle and poultry in the U.S. [113], in Alpacas at a poultry farm that was depopulated due to HPAI A(H5N1) in May 2024 in Idaho [115], and in swine at a backyard farm raising multiple species on 30 October 2024 [116]. This highlights the ever-changing nature of AIVs and the necessity for continued surveillance to monitor the evolution and potential threats to public health [106].

### 9.5. Birds and Humans: The Zoonotic Potential

Highly pathogenic avian influenza viruses of the H5N1 subtype initially emerged in southern China in 1996 and caused significant outbreaks in poultry in Hong Kong in 1997, leading to 18 human infections [117]. These human infections led to the first reported human death from A(H5N1), involving a 3-year-old child in Hong Kong, China, on 21 May 1997 [118]. Although the 1997 influenza A(H5N1) outbreak in poultry was controlled, the virus was not eradicated from this important population [117]. The virus re-emerged in 2003 and has since spread widely throughout Asia, followed by Africa, Europe, and the Middle East, causing numerous poultry outbreaks and sporadic human infections [117]. Overall, avian influenza viruses, primarily H5N1, H7N9, H5N6, and H9N2, have been responsible for over 2500 human infections worldwide since 2003 [5].

Humans can become infected with AIVs through direct contact, i.e., handling, slaughtering, culling, or processing of the infected animals, or indirectly through contaminated environments [118]. Swimming in contaminated water with infected bird feces may also pose a potential risk factor for humans contracting the disease [119]. Although foodborne transmission of the virus through consumption of properly cooked eggs or poultry has not been shown, human cases of A(H5N1) have been epidemiologically linked to the consumption of contaminated raw poultry products [118]. Depending on the virus strain and characteristics of susceptible humans, the exposure can cause mild, flu-like symptoms to severe, life-threatening infections [118].

Between 2021 and July 2024, 35 cases of A(H5N1) virus infections in humans were reported to the WHO, along with five detections of A(H5) viruses in individuals exposed to A(H5N1)-infected animals in the United States [120]. Of the 31 cases with known HA subtype, 17 cases belong to clade 2.3.4.4b [120]. The first human case of A(H5) clade 2.3.4.4b in the U.S. was detected in a poultry farm worker who was involved in culling in a poultry premise that was suspected of H5N1 in April 2022 in Colorado [89]. The second human case was associated with dairy cattle in Texas [121]. To date, there have been 66 human-reported cases of HPAI A(H5N1) clade 2.3.4.4 in the U.S. in 2024, with all but one case reporting contact with infected dairy cattle and/or poultry [122].

The symptoms typically reported in the patients were fatigue for a few days and conjunctivitis, followed by full recovery [89,121]. The CDC detected an E627K mutation in the PB2 gene in an H5N1 isolate from a patient in Kansas, suggestive of mammalian adaptation mutation [114,123], highlighting the rapid evolution of the virus and the potential for mammalian adaptation [105]. However, since similar substitutions have been observed in AIVs affecting humans and other mammalian hosts, as well as avian influenza viruses, such as H7N9, without affecting virus transmissibility, the CDC and USDA risk assessment of the HPAI for the general population remains low [95,107,121,124,125,126,127]. Recently, PB2 E627K, HA E186D, and Q222H, indicating mammalian adaptation mutations and enhanced receptor-binding capability to mammalian cell-surface receptors (i.e., α2,6 sialyloligosaccharides), were detected in a patient manifesting critical illness in Canada [128]. People with exposure to infected animals, i.e., infected birds, livestock, and other animals, and/or environments contaminated by infected animals are thought to be at higher risk [129].

Although the virus caused mild infections in most of the affected humans, bovine HPAI A(H5N1) clade 2.3.4.4b viruses isolated from the eye secretion of an infected farm worker were lethal in experimentally infected ferrets and mice [130]. The virus is transmitted efficiently via respiratory droplets among ferrets, and 83% of the exposed ferrets (n = 5) succumbed to infection [130]. These observations suggest that the bovine HPAI A(H5N1) clade 2.3.4.4b, which has efficient transmission via the respiratory route in ferret models, may have the ability to bind and replicate in the human upper respiratory tract [130], increasing the evolving risk to humans.

While the evidence for co-speciation mutations of the HPAI A(H5N1) clade 2.3.4.4b in mammalian hosts remains insufficient [2], and ongoing human-to-human transmission has not been observed [113,121,131,132], mink to mink [97], cattle to cattle [108], and ferret to ferret transmission [130] of the HPAI A(H5N1) clade 2.3.4.4b has raised global concerns related to human health [2]. HPAI A(H5N1) clade 2.3.4.4b, therefore, poses a significant public health concern worldwide due to its devastating impacts on the poultry industry, livestock, and wildlife populations, as well as its potential risk to humans [105]. A One Health approach to surveillance of the AIVs at the animal–human interface is thus required to tackle the ongoing threat and prevent a future pandemic [120].

## 10. Pandemic Potential

The three major human pandemics in the 20th century caused by influenza viruses were the 1918 pandemic of H1N1, thought to have originated in Spain; the 1957 pandemic of H2N2, believed to have originated in Asia; and the 1968 pandemic of H3N2, asserted to have originated in Hong Kong [133]. These pandemics are thought to have resulted from the reassortment of influenza strains circulating in avian and human hosts [70,133,134]. The Asian pandemic, Asian/57 (H2N2), kept five gene segments from the strain circulating in humans and acquired three gene segments (PB1, HA, and NA) from the avian gene pool circulating in wild ducks by reassortment [69]. The Hong Kong pandemic, Hong Kong/68 (H3N2), retained six gene segments from the influenza strain circulating in humans and acquired two gene segments (HA and PB1) from the avian strain circulating in wild ducks by reassortment [69]. The 1918 Spanish flu is thought to have emerged from an avian-like virus, not a reassortant of swine or human influenza A viruses [69]. However, this assertion is controversial. According to [134], the 1918 pandemic H1N1 virus was a reassortant strain from mammalian host viruses, i.e., humans and swine, that were circulating at least 2–15 years prior to the pandemic and not from a pure avian host.

For an influenza pandemic to occur, an efficient human-to-human transmission of the virus, combined with limited to no immunity against the virus, is required [118]. Given the ecological success of HPAI H5N1 in a variety of poultry and wild bird species, combined with frequent human incursions, it is plausible that this virus will be the source of the next pandemic in human populations [4,62]. Though, it is difficult to predict how, where, and when (this) pandemic will occur [118].

The emergence of pandemic strains through reassortment events in the segmented genome of influenza viruses of avian or mammalian hosts, often occurring several years prior to the onset of a pandemic, underscores the critical importance of early surveillance efforts [134]. These efforts, aimed at detecting and characterizing the precursor viral strains, are vital in preventing future pandemics by enabling timely interventions and preparedness [134]. Poor surveillance programs combined with weak biosecurity measures, on the other hand, may increase the risk of zoonotic exposure to HPAI from avian or mammal hosts, leading to the emergence of novel strains capable of maintaining a sustained transmission cycle between humans, posing a potential risk for the emergence of a future pandemic [2].

Currently, the primary emphasis of worldwide human influenza surveillance centers around the hemagglutinin (HA) gene of influenza, which is crucial for vaccine development, though it will not be adequate for early detection of an(y) emerging pandemic(s) [134]. In addition to characterizing all eight gene segments of viral isolates [134], there is a need to adopt a multi-jurisdictional and collaborative One Health approach in the surveillance of AIVs at the wild–domestic and human–domestic interfaces to better understand the evolution, adaptation, and spread of the virus, preventing the potential health and socioeconomic complications due to a possible pandemic [118,135].

## 11. Control and Mitigation Measures 

Vaccination and culling of infected populations are the two main strategies for preventing and controlling influenza [3,133]. However, such efforts in wild birds and mammals offer limited benefits. Instead, the focus should be on enhancing monitoring and surveillance programs, especially along migratory bird flyways, where wild birds congregate in large numbers and intermingle with other animal species, facilitating the spread and spillover of the virus [136].

With the ongoing HPAI A(H5) clade 2.3.4.4b outbreak in the U.S., concerns extend beyond the poultry population. The virus has evolved into a multi-host threat, impacting wildlife, livestock, and humans. The rapid and ongoing evolution of HPAI A(H5) clade 2.3.4.4b increases the potential for the virus to adapt to mammalian species, including humans [82], which could have devastating consequences for human health and the economy. In response to this animal and public health concern, control and mitigation strategies should prioritize preparedness, monitoring, surveillance, research, communication, and targeted management practices [135]. These efforts will help us better understand the virus’s ongoing evolution, determine the source of its emergence, manage outbreaks in at-risk host populations, and assess host tropism [87]. Again, a multi-jurisdictional One Health approach at the human–animal interface is crucial in addressing this threat [135].

At the individual level, especially for occupational risk mitigation, it is essential to use proper personal protective equipment (PPE) when working with infected animals or in contaminated environments [95]. As this topic is beyond the scope of the current review, we recommend referring to the following sources for more detailed information: [120,125,137].

## 12. Conclusions and Future Directions

Avian influenza viruses (AIVs) have a segmented genome consisting of eight negative-sense, single-stranded RNA segments. These segments encode 10 key viral proteins: PB2, PB1, PA, and NP, which are responsible for RNA replication; NS1, which helps the virus evade the host immune response; M1, which maintains the structural integrity of the virus particle; NEP, which facilitates the nuclear export of vRNPs; and HA, M2, and NA, which mediate binding, uncoating, and subsequent spread of the virion, respectively.

AIVs exhibit a high propensity for both genetic drift and genetic shift, with the latter primarily responsible for the extensive diversity of virus subtypes, continued evolution, and broad host tropism through reassortment. AIVs are classified into low pathogenic (LPAI) and high pathogenic (HPAI) strains, with HPAI strains posing the highest risks to animal and human health. Over the past two decades, highly pathogenic subtypes of avian influenza viruses have led to the death or culling of over 547 million poultry and caused significant morbidity and mortality among various wildlife populations, with spillover effects into livestock and humans. The World Health Organization (WHO) has documented over 878 human cases associated with HPAI viruses, with a 52% case fatality rate reported across 23 countries during the last two decades.

As a global concern, the threat of highly pathogenic avian influenza (HPAI) is mainly associated with the H5 gs/GD viruses, which were first detected in China in 1996. Since their discovery, these viruses have undergone extensive reassortment, evolving into 10 clades and multiple subclades. During an outbreak in 2014, clade 2.3.4.4c viruses of the gs/GD lineage led to the death or culling of over 50 million poultry in the U.S. In late 2021, the first outbreak of Eurasian-lineage gs/GD HPAI A(H5N1), belonging to clade 2.3.4.4b, was detected in the U.S. through the active surveillance of hunter-harvested waterfowl in South and North Carolina. These viruses were introduced into the U.S. through the Atlantic flyway, facilitated by the intercontinental movement of migratory birds.

The ongoing outbreak has had devastating consequences for the U.S. poultry industry, wildlife, and livestock, with increased spillover into the human population. Although the virus causes mild infections in humans, some bovine viruses isolated from human patients have shown the ability to efficiently spread among animal models, causing extreme pathogenicity and death. The threat is now widespread, posing risks not only to numerous wild and domestic animal species but also to human health and international trade in food animal products.

Waterfowl, gulls, and shorebirds are natural reservoirs of AIVs, facilitating the continued evolution, spread, and persistence of these viruses through long-distance migrations, which often span continents. These movements promote the spread and spillover of AIVs both within and between species, as well as across countries and continents, supporting the sustained evolution and host adaptation of AIVs. While there are host- and virus-specific barriers that could hinder AIVs’ ability to adapt to and infect new mammalian hosts, antigenic shifts—primarily due to reassortment of AIV gene segments during co-infections with different influenza A viruses (IAVs)—can lead to the acquisition of markers necessary for enhanced polymerase activity and evasion of host immune surveillance.

For example, RIG-I and MxA sequester avian influenza vRNPs in the cytoplasm, preventing their nuclear import for transcription and replication. However, mutations such as the shift from glutamic acid to lysine at position PB2 627 (E627K), combined with changes in NP, enable AIVs to evade detection by RIG-I and binding by MxA. While AIVs with the 627E PB2 are susceptible to antiviral signaling proteins like MAVS, those with the 627K PB2 mutation can bypass this barrier. As a result, AIVs have established a broad host range, affecting a variety of mammalian hosts, including humans, horses, felids, minks, marine mammals, wild mammals, and livestock.

For influenza virus to cause a human pandemic, an ongoing human-to-human transmission of the virus, which is a primary precursor, coupled with limited or no human immunity to the virus, is required. Due to the ecological success of the currently circulating HAPI A(H5N1) clade 2.3.4.4b circulating among a variety of birds and mammalian species, alongside the heightened human spillovers, it is likely that these viruses could be the source of a next pandemic. Nevertheless, we cannot predict when, how, and where it may occur. Global efforts are, thereby, warranted to curtail the current outbreaks and prepare for a potential pandemic.

The human-to-human transmission of HPAI A(H5N1) clade 2.3.4.4b has not been documented, suggesting a low risk to the general population. However, the detection of the mammalian adaptation mutations in two patients (one in Canada and the other in the U.S.), the efficient transmission of the reassortant Eurasian/American HPAI A(H5N1) clade 2.3.4.4b viruses among minks, cows, from cows to humans, and from poultry to humans, combined by the respiratory spread leading to extreme pathogenicity and death in animal models, have raised global concerns regarding the virus’s potential to successfully adapt to humans, potentially causing widespread infections. The influenza pandemics of 1918, 1957, and 1968, thought to have emerged through reassortments between avian- and mammalian-origin influenza A viruses, serve as a reminder of how these viruses can suddenly emerge as global health threats, affecting humans worldwide. It is crucial to closely monitor the currently circulating HPAI A(H5N1) clade 2.3.4.4b viruses within their natural reservoirs, as well as in infected livestock and humans, to track the virus’s evolution and spread.

To enhance management and mitigation efforts, a whole-genome sequencing approach should be pursued to assess and analyze all eight gene segments of the virus. This strategy not only allows for the identification of mammalian adaptation and antiviral resistance markers but also enables the detection and characterization of novel strains that may circumvent host barriers. Given the potential for unexpected AIV host jumps and their ability to infect a variety of mammalian species with efficient horizontal transfer, it is vital to align research priorities with pandemic preparedness efforts.

Therefore, in addition to strengthening surveillance efforts in the natural reservoirs of AIVs, it is reasonable to vigilantly monitor newly infected mammalian hosts, such as dairy cattle and swine, which share extensive time and space with humans, particularly in settings like exhibition shows. Monitoring these species may allow for early detection and characterization of viral strains that pose significant risks to human health due to their potential for widespread infections. Furthermore, experimental animal challenge studies with novel strains should be conducted to deepen our understanding of viral kinetics in mammalian hosts and to monitor emerging risks to both animals and humans.

Achieving these goals requires sustained surveillance efforts involving a multi-jurisdictional One Health approach that integrates humans, animals, and shared environments. Such an approach is essential for managing and mitigating current outbreaks, as well as for better preparing for potential future pandemics.

## Data Availability

Data are contained within the article.
